# Longitudinal transcriptomic analysis of mucosa in ulcerative colitis after anti-tumor necrosis factor withdrawal compared to continued treatment

**DOI:** 10.1093/ecco-jcc/jjag121

**Published:** 2026-08-12

**Authors:** Emma Rapp, Ingrid Prytz Berset, Sinan U Umu, Espen Sønderaal Bækkevold, Karen Sivertsen Utheim, Alexander Dietrich, Markus List, Kjersti Thorvaldsen Hagen, Frode Lerang, Dag Arne Lihaug Hoff, Knut E A Lundin, Frode Lars Jahnsen, Diana Domanska

**Affiliations:** Department of Pathology, Oslo University Hospital, 0372 Oslo, Norway; Institute of Clinical Medicine, University of Oslo, 0450 Oslo, Norway; Department of Gastroenterology, Møre & Romsdal Hospital Trust, 6017 Ålesund, Norway; Norwegian Coeliac Disease Research Centre, University of Oslo, 0372 Oslo, Norway; Department of Pathology, Oslo University Hospital, 0372 Oslo, Norway; Institute of Clinical Medicine, University of Oslo, 0450 Oslo, Norway; Department of Pathology, Oslo University Hospital, 0372 Oslo, Norway; Institute of Oral Biology, University of Oslo, 0372 Oslo, Norway; Institute of Clinical Medicine, University of Oslo, 0450 Oslo, Norway; Data Science in Systems Biology, TUM School of Life Sciences, Technical University of Munich, 85354 Freising, Germany; Data Science in Systems Biology, TUM School of Life Sciences, Technical University of Munich, 85354 Freising, Germany; Munich Data Science Institute (MDSI), Technical University of Munich, 85748 Garching, Germany; Institute of Clinical Medicine, University of Oslo, 0450 Oslo, Norway; Department of Gastroenterology, Østfold Hospital Trust, 1714 Grålum, Sarpsborg, Norway; Department of Gastroenterology, Møre & Romsdal Hospital Trust, 6017 Ålesund, Norway; Department of Health Sciences, Norwegian University of Science and Technology, 6009 Ålesund, Norway; Institute of Clinical Medicine, University of Oslo, 0450 Oslo, Norway; Norwegian Coeliac Disease Research Centre, University of Oslo, 0372 Oslo, Norway; Department of Gastroenterology, Oslo University Hospital, Rikshospitalet, 0372 Oslo, Norway; Department of Pathology, Oslo University Hospital, 0372 Oslo, Norway; Institute of Clinical Medicine, University of Oslo, 0450 Oslo, Norway; Department of Pathology, Oslo University Hospital, 0372 Oslo, Norway; Faculty of Mathematics and Computer Science, University of Warmia and Mazury in Olsztyn, 10-710 Olsztyn, Poland

**Keywords:** ulcerative colitis, anti-TNF withdrawal, gene expression

## Abstract

**Background and Aims:**

Monoclonal antibodies against tumor necrosis factor (anti-TNF) are effective agents in the treatment of moderate-to-severe ulcerative colitis (UC). It is unclear whether such treatment can safely be discontinued without relapse. This study aimed to identify biomarkers to predict which patients can successfully stop anti-TNF treatment, and to increase our understanding of the pathogenesis of relapse after withdrawal and flare on continued therapy.

**Methods:**

We analyzed longitudinal bulk RNA-sequencing data derived from rectal mucosal biopsies from 163 patients in the BIOSTOP study collected at baseline, after 2 years (end of study), and at flares or relapse points.

**Results:**

In this cohort, pre-withdrawal transcriptomic profiles did not distinguish patients who later relapsed from those who remained in long-term remission. Longitudinal analysis of patients who relapsed or flared were both characterized by inflammatory gene expression profiles (eg, CXCL9, CXCL10, OSMR, SELE) and changes in cell-type composition typical for active UC (eg, monocytes/macrophages and *THY1^+^ FAP^+^ PDPN^+^* activated fibroblasts). Both groups showed enrichment of T-cell-associated transcriptional signatures, including CD8 T-cell-related genes and IL-17-associated pathways. Additionally, both patient groups showed increased expression of targets of alternative therapeutic agents, suggesting potential treatment options for specific patient subgroups.

**Conclusions:**

Our findings provide insights into the inflammatory mechanisms driving relapse after anti-TNF withdrawal and flare on continued treatment. The dataset offers a valuable resource for future research to improve personalized treatment strategies in UC.

## 1. Introduction

Ulcerative colitis (UC) is a chronic inflammatory bowel disease (IBD) with a global prevalence of around 5 million individuals. UC is particularly common in Norway and other Nordic countries.^1^ Common symptoms include abdominal cramping, diarrhea, and rectal bleeding, which often result in a reduced quality of life. UC is also associated with an increased risk of disease-related complications, including colorectal cancer.^1–3^

UC affects the mucosa of the large intestine (colon and rectum) and is characterized by a complex inflammatory reaction with infiltration of large numbers of innate (eg, macrophages and neutrophils) and adaptive (eg, T cells, B cells, and plasma cells) immune cells. Current treatment options include biologics and small molecules that inhibit immune cell trafficking and cytokine production.

Inhibition of the cytokine tumor necrosis factor (anti-TNF therapy) has been shown to be effective in the treatment of moderate-to-severe UC.^4^^,^^5^ However, long-term use of anti-TNF may be associated with an increased risk of infection, malignancies, and allergic reactions.^6^ Long-term use may further induce immunological disease alteration and immunogenicity, both associated with loss of response and discontinuation of the treatment.^7^ In addition, although biosimilar drugs have reached the market, the cost of treatment is still considerable.^8^^,^^9^ However, properly performed randomized, controlled trials on discontinuation are scarce.^10^^,^^11^

The BIOSTOP study is a clinical trial evaluating the outcomes following anti-TNF withdrawal in UC patients in clinical, biochemical and endoscopic remission.^12^ The study included 163 UC patients who were randomized to either withdrawal or continuation of anti-TNF therapy. After a follow-up period of 2 years, 52% of patients who discontinued anti-TNF therapy experienced relapse, compared to 12% in the maintenance group. These findings are consistent with results from other clinical trials.^13–17^ Importantly, three out of four patients in the BIOSTOP withdrawal group who resumed anti-TNF therapy regained both clinical and endoscopic remission. Together, these findings suggest that anti-TNF withdrawal may be considered in selected patients.

However, there are currently no biomarkers available to predict the clinical outcomes following anti-TNF withdrawal. In this study we analyzed bulk RNA-sequencing (RNA-seq) data derived from mucosal biopsies collected at baseline, at the time of relapse in both groups, and at the 2-year follow-up in the patients who remained in long-term remission. The aim was to identify biomarkers predictive of clinical outcome after anti-TNF withdrawal and to increase our understanding of the inflammatory processes underlying relapse after treatment withdrawal or flare on continued therapy.

## 2. Material and methods

### 2.1. Patient cohort and tissue samples

The BIOSTOP trial is a Norwegian, multicenter prospective, randomized, controlled, and open-label clinical study designed to evaluate outcomes following discontinuation of anti-TNF therapy in patients with UC.^12^ A total of 163 patients were included in the per-protocol population: 82 patients were randomized to anti-TNF withdrawal, and 81 to continued treatment. All patients had a confirmed UC diagnosis and had received continuous anti-TNF therapy for at least 1 year prior to inclusion. Additional inclusion criteria included clinical remission for a minimum of 3 months, two consecutive fecal calprotectin measurements <200 µg/g, and a Mayo endoscopic score (MES) of 0-1 to ensure that the patients were in deep clinical, biochemical, and endoscopic remission at inclusion.

Patients were followed for 2 years, with assessment of clinical disease activity every 3 months. Monitoring included fecal calprotectin, serum biomarkers, and measurements of anti-TNF drug and antibody levels. Mucosal biopsies were obtained from all colonic segments (ascendens, transversum, descendens, sigmoid, and rectum) at baseline and after the 2-year follow-up. Additional biopsies were collected at the time of suspected relapse or flare. These unscheduled biopsies were obtained following the emergence of clinical signs or symptoms, typically defined as 6-point Mayo score >1 and/or elevated fecal calprotectin levels >200 µg/g. UC relapses were confirmed endoscopically by MES > 1. Written informed consent was obtained from all study participants, and the clinical study followed Helsinki guidelines and was approved by the Regional Committee for Medical Research Ethics (REK Midt 2016/531). All biopsy specimens were deposited in the general biobank “Tarmsykdommer” (REK Sør-Øst #20521), where all study participants have signed a separate informed consent.

Mucosal biopsies designated for transcriptomic analysis were immediately immersed in RNAprotect Cell Reagent (Qiagen: Cat. No.: 76526) and fresh frozen at −80 °C for bulk mRNA sequencing. Additional biopsies were formalin-fixed and processed for histologic examination. Histologic inflammatory activity was assessed using the Robarts Histology Index (RHI), a validated scoring system for UC.^18^ The RHI score evaluates four components: chronic inflammatory infiltrate level, lamina propria neutrophils, neutrophils in epithelium, and the presence of erosion or ulceration. Each component is scored individually, and the sum yields a total RHI score ranging from 0 (no histologic disease activity) to 33 (severe histologic disease activity).

### 2.2. Tissue dissociation and RNA isolation

Frozen rectal biopsies were placed in a tube containing 1 mL TRIzol reagent® (InvitrogenTM: Cat. No.: 15596026) and ¼-inch ceramic beads as lysis matrix (MP biomedicals: Cat. No.: 6540-424). Tissue dissociation was performed using a FastPrep®-24 (MP biomedicals) with three 30-s shaking cycles at 6.0 m/s with samples resting on ice for 2-2-5 min between cycles. Following tissue lysis, RNA was purified using the RNeasy Mini kit (Qiagen: Cat. No.: 74106), according to the manufacturer’s protocol. Purified RNA was stored at −80 °C and sent to Novogene (UK) for downstream processing. RNA quantity, integrity, and purity were assessed using the Agilent 5400 Fragment Analyzer System (Agilent Technologies). Only samples meeting the quality thresholds defined by the sequencing center were included for sequencing.

### 2.3. Library preparation and bulk mRNA-seq

Library preparation and sequencing were performed by Novogene (UK). Libraries were prepared using the Novogene NGS Stranded RNA Library Prep Set (PT044). mRNA was isolated from 800 ng of total RNA using poly-T oligo-attached magnetic beads, followed by fragmentation and synthesis of the first-strand cDNA using random hexamer primers. Second-strand cDNA synthesis was performed using dUTPs. Subsequent steps included end repair, A-tailing, adapter ligation, size selection, USER enzyme digestion, amplification, and purification. Library quantification was performed using Qubit and real-time PCR, and fragment size distribution was assessed using a Bioanalyzer. Libraries were pooled and sequenced on an Illumina NovaSeq 6000 platform using paired-end 150-bp reads (PE150), generating approximately 30 million read pairs per sample (∼9 Gb of raw data) (Novogene).

### 2.4. RNA-seq data processing

Bulk RNA-seq data were processed using the publicly available nf-core/rnaseq pipeline (v.3.12.0), which is a scalable, robust, and reproducible workflow implemented in Nextflow (v.22.04.0).^19^ This pipeline includes comprehensive quality control (QC), alignment and transcript quantification, following current best practices for reproducible and standardized RNA-seq data analysis. Key steps included adapter trimming with Trim Galore (v.0.6.7),^20^ alignment to the reference genome using the STAR aligner (v.2.6.1d),^21^ and transcript quantification with Salmon (v.1.5.2).^22^ The default pipeline uses genomes provided by AWS iGenomes (https://ewels.github.io/AWS-iGenomes/), and the NCBI GRCh38 human reference genome was chosen for this analysis. RAW count matrices generated by the pipeline were used for downstream differential expression analysis.

### 2.5. Differential gene expression analysis

Samples with duplicate read rates exceeding 70% were excluded, as high duplication rates may indicate low library complexity or PCR over-amplification. After quality control filtering, samples from 154 patients were retained for downstream analyses (withdrawal group: remission = 38, relapse = 40; maintenance: remission = 66, flare = 10).

Differential gene expression (DEG) analysis was performed using the DESeq2 package (v.1.42.1)^23^ in R (v.4.3.1), using raw counts and sample metadata (Table S1) as input. Principal component analysis (PCA) was conducted to explore clustering by clinical (eg, disease extent, concomitant medication use, age, gender, and disease activity scores) or technical variables across the full dataset and within the different subsets. No strong clustering was observed for most variables in the subsets; a modest clustering by gender was observed and therefore gender was included as a covariate in the baseline comparison (Figure S1). Prior to constructing DESeq2 objects, data were filtered according to condition, time point, or treatment group to include only relevant samples for each comparison.

Baseline differential gene expression analyses were performed using the design formula ∼ Gender + Condition. Longitudinal pairwise comparisons were performed using a paired design formula ∼ PtID + Timepoint, where patient ID was included in the design to account for within-patient variability over time. DESeq2’s default normalization and dispersion estimation procedures were applied. Low count genes were filtered by retaining genes with at least 10 total counts across samples. Log_2_ fold changes were shrunk using the lfcShrink function to improve estimates for genes with low counts or high variability.^24^ Genes were considered significantly differentially expressed if the adjusted *P*-value was <.05 and the absolute log_2_ fold change exceeded 1. Variance-stabilizing transformation (VST) normalization was used for all gene expression visualizations.

### 2.6. Pathway enrichment analysis

Pathway enrichment analysis was performed using the clusterProfiler package (v.4.10.1)^25^ in R (v.4.3.1). Databases used included Kyoto Encyclopedia of genes and genomes (KEGG),^26^ Gene ontology (GO),^27^^,^^28^ and Reactome.^29^ GO analysis covered Biological Process, Cellular Component, and Molecular Function categories. All significantly differentially expressed genes from DEG analysis were used as input, and pathways with an adjusted *P* value of <.05 were considered significant. 

### 2.7. Cell-type deconvolution

Cell-type composition was estimated from bulk mRNA-seq data using the omnideconv package (v.0.1.1)^30^ in R (v.4.3.1). A publicly available human single-cell RNA-seq atlas of inflamed and non-inflamed intestinal tissue from patients with IBD (987 743 cells),^31^ was used as input for omnideconv to learn cell-type-specific gene signatures. This dataset provides high-resolution profiling of mucosal immune cell populations and is thus well suited as a reference to infer cellular composition in rectal biopsies from UC patients.

The single-cell dataset was pre-processed using scanpy^32^ (v.1.10.4) and Seurat^33^ (v.5.3.0). Prior to quality control, only rectal biopsies from UC patients post anti-TNF therapy were retained for downstream analysis. Quality control of the single-cell reference was performed according to current best practices for single-cell RNA-seq analysis.^34^ Cells were filtered based on three metrics: total counts, number of detected genes, and fraction of mitochondrial reads. Outliers were identified using the median absolute deviation (MAD) method, applying thresholds of 5 MADs for total counts, and detected genes, and 3 MADs for mitochondrial content. Low-quality cells exceeding these thresholds were removed. Cell type annotations were assigned based on the “major” annotation column in the reference dataset metadata. To reduce computational loads while preserving representative profiles, the dataset was further subsampled to a maximum of 200 high-quality cells per cell type to construct the signature matrix.

Transcript-per-million (TPM) normalized counts from bulk mRNA-seq data, the filtered and subsampled single-cell reference, and sample metadata were used as input to the Dampened Weighted Least Squares (DWLS) algorithm,^35^ implemented via omnideconv with default parameters. Among the 11 tools included in omnideconv, DWLS was selected as it performed consistently well in recent benchmarks.^30^ Estimated immune cell fractions were compared longitudinally within the clinical groups. Differences in estimated cell proportions were assessed using two-sided Wilcoxon rank-sum tests, and *P*-values were adjusted for multiple testing using the Benjamini–Hochberg (BH) false discovery rate (FDR) method.

## 3. Results

### 3.1. Clinical outcomes in the BIOSTOP cohort

A total of 163 patients were included in the BIOSTOP trial per-protocol analyses; 82 patients were randomized to anti-TNF withdrawal and 81 to continued treatment. During the 2-year follow-up, 44 patients (54%) in the withdrawal group experienced clinical relapse, with a median time to relapse of 8.6 months after treatment discontinuation. In the maintenance group, 10 patients (12%) experienced flare, with a median time of 13.3 months after inclusion.^12^

### 3.2. Gene expression at baseline does not predict relapse after anti-TNF discontinuation

**Figure 1.**
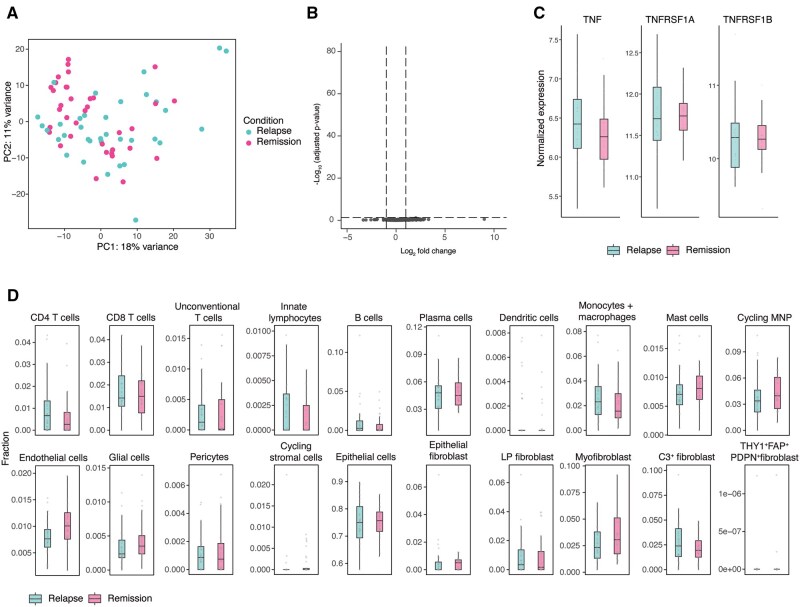
Baseline transcriptomic profiles do not distinguish remission from relapse after anti-tumor necrosis factor (anti-TNF) withdrawal. (A) Principal components analysis plot of baseline biopsies from patients who maintained long-term remission versus those who relapsed after anti-TNF withdrawal (remission = 36, relapse = 36). (B) Differential gene expression analysis comparing remission vs relapse at baseline within the withdrawal group (same number of individuals as in A). (C) Normalized expression of *TNF*, *TNFRSF1A*, and *TNFRSF1B* at baseline in the withdrawal group. (D) DWLS deconvolution estimates (TAURUS reference) comparing relapse versus remission at baseline in the treatment withdrawal group.

To assess whether gene expression profiles at baseline could predict relapse following anti-TNF withdrawal, we compared bulk RNA-seq data derived from baseline rectal biopsies between the patients who later relapsed and those who remained in remission. Unsupervised PCA of the transcriptomic profiles revealed substantial overlap between the two groups (Figure 1A), and differential expression analysis identified no significant DEGs (Figure 1B). Notably, expression of *TNF* and its receptors (*TNFRSF1A* and *TNFRSF1B*) did not differ between groups (Figure 1C).

To estimate the relative abundance of mucosal cell populations, we performed cell-type deconvolution of bulk RNA-seq data using the omnideconv package,^30^ with an annotated single-cell RNA-seq atlas of both inflamed and non-inflamed intestine from patients with IBD as reference.^31^ This analysis resolved 21 distinct cell phenotypes, including several subsets of immune cells, stromal cells, endothelial cells, and epithelial cells (Figure 1D). The relative fractions were not significantly different for any of the cell subsets between the two groups at baseline (Figure 1D). Together, these results indicate that neither baseline gene expression nor estimated mucosal cell composition distinguished patients who later relapsed from those who maintained remission after anti-TNF withdrawal.

### 3.3. Relapse after anti-TNF withdrawal is associated with a strong innate immune response

**Figure 2.**
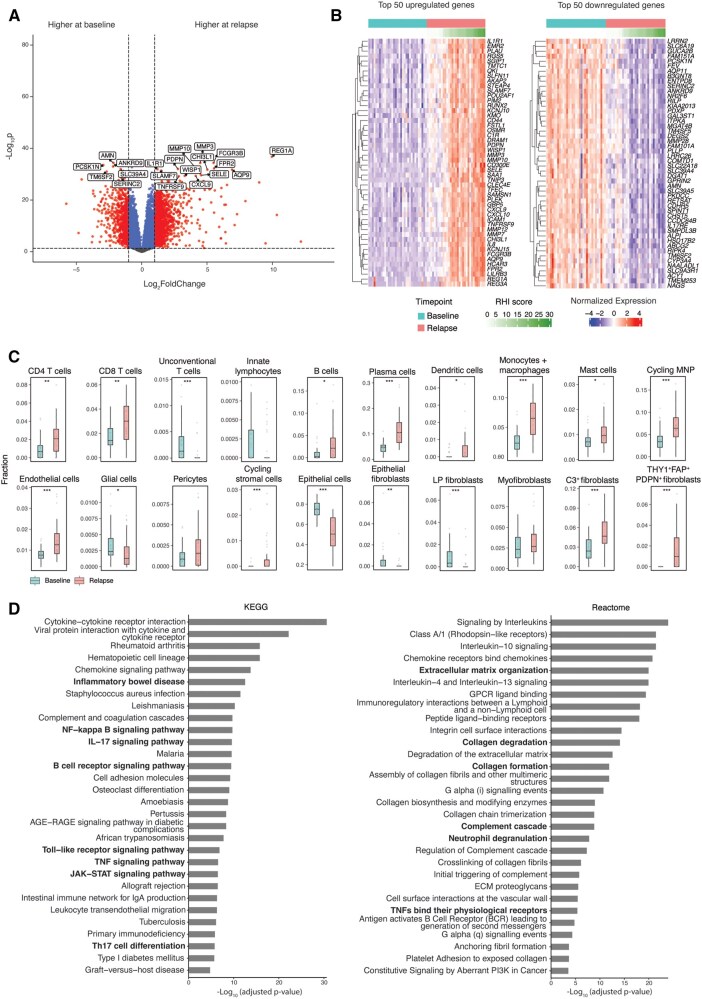
Ulcerative colitis (UC) patients who relapse after anti-tumor necrosis factor (anti-TNF) withdrawal are associated with a strong innate immune reaction. (A) Paired differential gene expression (DEG) analysis comparing baseline to relapse in the withdrawal group (*n* = 33). Genes with positive log_2_FoldChange are upregulated at point of relapse compared to baseline. (B) Heatmaps of the top 50 upregulated and downregulated DEGs ranked by BH-adjusted *P*-values, with hierarchical gene clustering. (C) DWLS deconvolution estimates (TAURUS reference) comparing baseline versus relapse. Significance is indicated by BH-adjusted *P*-values (*P-adjusted < .05, **P-adjusted < .01, ***P-adjusted < .001). (D) KEGG and Reactome pathway enrichment of significantly upregulated genes in relapse (BH-adjusted *P*-value < .05), emphasizing pathways implicated in UC.

To characterize transcriptomic changes associated with relapse in the withdrawal group, we analyzed rectal biopsies collected longitudinally. In the patients who relapsed, we identified 1546 upregulated and 878 downregulated genes at time of relapse compared with baseline (adjusted *P* < .05 and |log_2_FoldChange| > 1) (Figure 2A). Among the most significant upregulated genes, many were inflammatory mediators and tissue remodeling factors (eg, *CXCL9*, *CXCL10*, *CHI3L1*, *FPR2*, *MMP3*, *MMP7*, *MMP10*, *MMP12*, *OSMR*, *PDPN*, *REG1A*, *REG3A*, *SELE*) (Figure 2B). These genes are mainly expressed by myeloid and structural cell types, suggesting prominent involvement of macrophages, neutrophils, and stromal and epithelial cells. Consistently, deconvolution analysis showed significantly increased relative fractions of cycling mononuclear phagocytes (MNPs) and monocytes/macrophages at relapse (Figure 2C). Endothelial cells were also increased, while fibroblast subsets showed divergent behavior: *THY1^+^ FAP^+^ PDPN^+^* fibroblasts and *C3^ +^* fibroblasts increased, whereas epithelial-associated fibroblasts decreased. Interestingly, high numbers of *THY1^+^ FAP^+^ PDPN^+^* activated fibroblasts have been found in active UC,^31^ and accumulation of this cell type has been associated with non-responsiveness to anti-TNF treatment.^36^ Pathway enrichment analysis using KEGG and Reactome databases showed significant enrichment of pathways associated with innate immune pathways, including NF-kappa B signaling, Toll-like receptor signaling, complement cascade activation, and neutrophil degranulation. Pathways linked to stromal activation and matrix remodeling such as extracellular matrix organization, collagen formation, and degradation, were also enriched (Figure 2D).^27–29^

In addition, several adaptive immune cell populations (CD4 T cells, CD8 T cells, B cells, plasma cells, and DCs) were increased at relapse (Figure 2C), and pathways including IL-17 signaling and B cell receptor signaling were enriched (Figure 2D), indicating concurrent activation of adaptive immunity during the inflammatory reaction.

**Figure 3.**
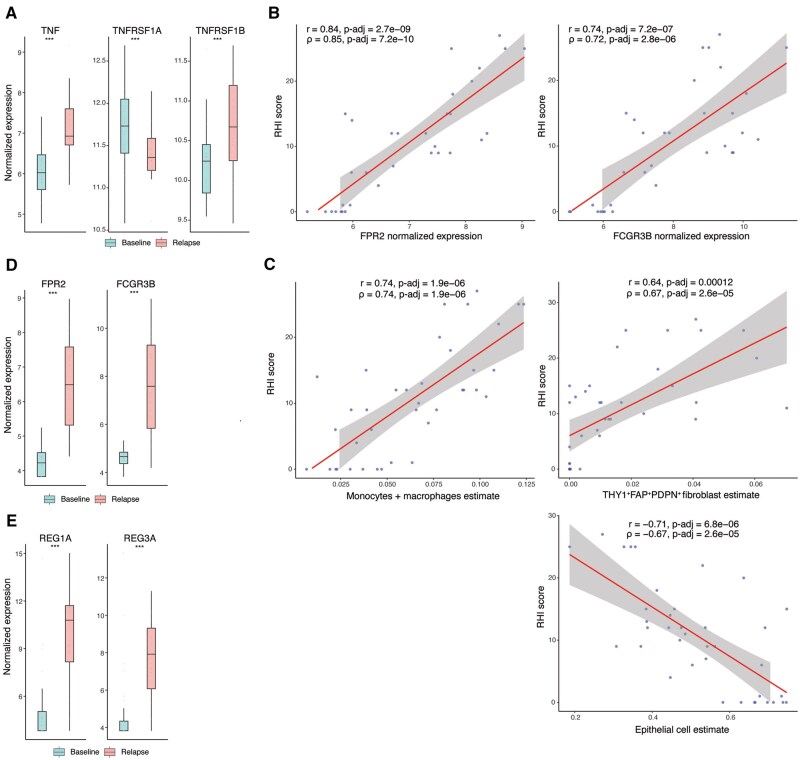
Expression of key genes, and correlation with RHI. (A) Variance-stabilizing transformation (VST)-normalized expression of *TNF*, *TNFRSF1A*, and *TNFRSF1B* at baseline and relapse in the treatment withdrawal group (same samples as Figure 2). Statistical significance is indicated by BH-adjusted *P*-values (*P-adjusted < .05, **P-adjusted < .01, ***P-adjusted < .001). (B) Correlation between neutrophil markers (*FPR2* and *FCGR3B*) and RHI. (C) Correlation between selected cell-type estimates (epithelial cells, monocytes/macrophages, *THY1^+^ FAP^+^ PDPN^+^* fibroblasts) and RHI. (D) Expression of *FPR2* and *FCGR3B* at baseline versus relapse in the anti-TNF withdrawal group. (E) Expression of *REG1A* and *REG3A* at baseline versus relapse.

Because anti-TNF was discontinued in the withdrawal group, we next examined whether *TNF* expression was increased at relapse. Both *TNF* and the TNF receptor *TNFRSF1B* were significantly upregulated, whereas the receptor *TNFRSF1A* was downregulated (Figure 3A). We also found enrichment of the pathways “TNF signaling” and “TNFs bind to their receptors” (Figure 2D). Clinically, the observation that most relapsing patients regained clinical and endoscopic remission after reintroduction of anti-TNF further supports a central role for TNF in the inflammatory reaction at relapse.^37^

Histologic assessment using RHI showed that most patients who relapsed had RHI ≥ 6, consistent with histology-verified inflammation. As RHI is strongly influenced by infiltration of neutrophils,^18^ we asked whether bulk mRNA-seq captured neutrophil-associated signals. Expression of neutrophil-associated markers *FPR2* and *FCGR3B* (Figure 2B) correlated strongly with RHI (Figure 3B), indicating concordance between transcriptomic and histologic inflammation. Moreover, RHI correlated with changes in several estimated cell populations (Figure 3 and Figure S2a), particularly monocytes/macrophages and *THY1^+^ FAP^+^ PDPN^+^* fibroblasts (Figure 3C), while epithelial cell proportions showed an inverse relationship, consistent with immune infiltration reducing the relative epithelial fraction. Increased expression of *REG1A* and *REG3A* (Figure 3E) was compatible with Paneth cell metaplasia and suggested that the epithelium was actively involved in the inflammatory process.

### 3.4. Sustained remission after anti-TNF withdrawal is associated with a stable transcriptomic profile

Among the patients who remained in sustained remission after anti-TNF withdrawal (*n* = 34), only 170 significant DEGs were found when comparing samples from the 2-year follow-up to baseline (adjusted *P* < .05 and |log_2_FoldChange| > 1), of which 128 genes were upregulated and 42 genes were downregulated (Figure S3a). The most significant upregulated genes included several non-coding RNAs, less-characterized transcripts, and a subset of immune-related genes such as *CXCL9*, *CXCL10*, *IL8*, *IL6*, and *TNIP3* (Figure S3b). However, patients with increased expression of inflammatory genes also exhibited a positive RHI (Figure S3b), suggesting subclinical histologic activity in a subset of samples. Deconvolution analysis showed no significant differences in relative fractions of cell types between baseline and 2-year follow-up (Figure S3c).

### 3.5. Flare on continued anti-TNF therapy shows mixed innate and adaptive immune activation

**Figure 4.**
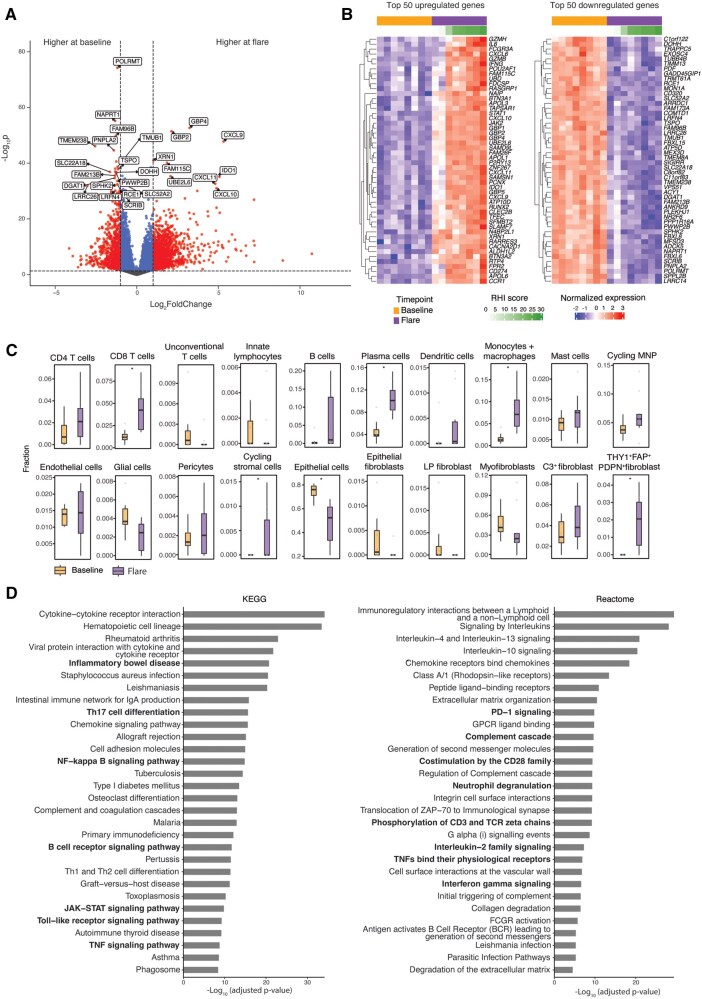
Ulcerative colitis (UC) patients who experience flare on continued anti-tumor necrosis factor (anti-TNF) therapy are associated with a stronger T cell-mediated profile. (A) Paired differential gene expression (DEG) analysis comparing baseline to point of flare in patients who continue anti-TNF treatment (*n* = 8). The genes with positive log_2_FoldChange are upregulated at flare compared to baseline. (B) Heatmaps of the top 50 upregulated and downregulated DEGs based on BH-adjusted *P*-values, with hierarchical clustering of genes. (C) DWLS deconvolution estimates (TAURUS reference) comparing baseline versus flare. Statistical significance is indicated by BH-adjusted *P*-values (*P-adjusted < .05, **P-adjusted < .01, ***P-adjusted < .001). (D) KEGG and Reactome pathway enrichment results for significantly upregulated genes in flare (adjusted *P*-value < .05), where relevant pathways for UC are highlighted.

**Figure 5.**
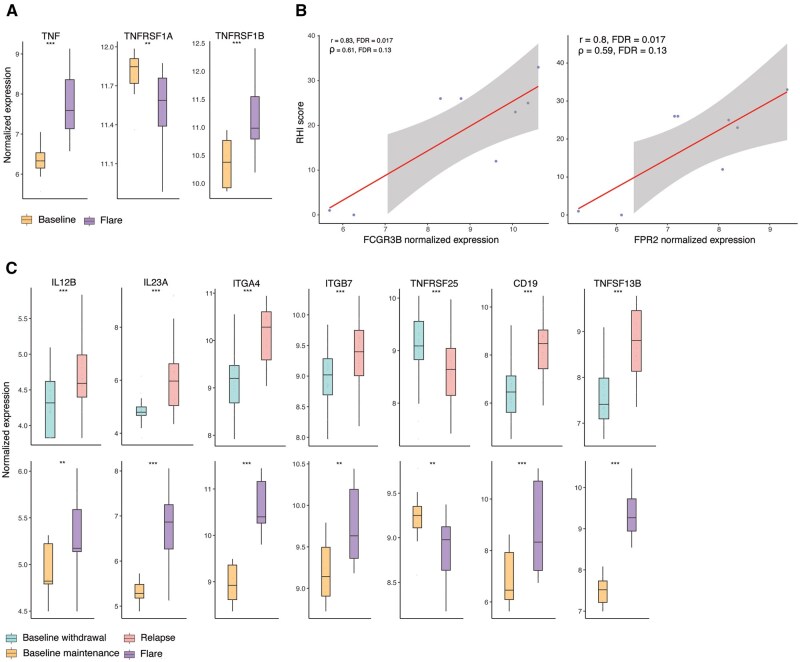
Expression of key genes and correlation with RHI in the maintenance group, and expression of alternative therapeutic targets in the maintenance and withdrawal groups. (A) Expression of *TNF*, *TNFRSF1A*, and *TNFRSF1B* in flare samples. (B) Correlation between RHI in the flare biopsies and *FCGR3B* and *FPR2* expression. (C) Longitudinal expression of selected therapeutic target genes: *IL12B*, *IL23A*, *ITGA4*, *ITGB7*, *TNFRSF25*, *CD19*, and *TNFSF13B* in relapse and flare groups.

Of the 81 patients who continued anti-TNF therapy, 10 experienced loss of response, despite lacking measurable anti-drug antibodies. Comparing baseline to the time of flare we identified 1653 upregulated and 1064 downregulated genes (adjusted *P* < .05 and |log_2_FoldChange| > 1) (Figure 4A). Several of the top upregulated genes overlapped with those observed at relapse after withdrawal (eg, *CXCL9*, *CXCL10*, *CXCL11*, *FPR2*, *SLAMF7*) (Figure 4B). However, many strongly induced genes were associated with T cell responses such as *IFNG*, *STAT1*, *GZMB*, *GZMH*, and *CD274* (Figure 4B). Consistently, deconvolution analysis showed a significant increase in CD8 T cells (Figure 4C) and pathway enrichment highlighted multiple T cell-associated pathways, including interferon signaling, CD28-family co-stimulation, phosphorylation of CD3 and TCRzeta chains, Interleukin-2 family signaling, and PD-1 signaling. Similar to the relapse in the withdrawal group, monocytes/macrophages, plasma cells, and *THY1^+^ FAP^+^ PDPN^+^* fibroblasts were significantly increased, together with enrichment of pathways linked to their functions (eg, NF-kappa B signaling, Toll-like receptor signaling, complement cascade, neutrophil degranulation, and B cell receptor signaling) (Figure 4D). RHI correlated strongly with *FCGR3B* and *FPR2* expression (Figure 5B), and enrichment of the neutrophil degranulation pathway further support the presence of neutrophil-associated inflammation.

*TNF* and *TNFRSF1B* were also significantly increased at flare, while *TNFRSF1A* was decreased (Figure 5A), consistent with enrichment of TNF signaling pathways (Figure 4D). Clinically, patients in the maintenance group with flares responded well when anti-TNF treatment was optimized, supporting the notion that TNF contributed to the inflammatory reaction.^12^

### 3.6. Long-term remission on maintenance therapy is associated with transcriptomic stability

Among the patients who remained in sustained remission while continuing anti-TNF therapy (*n* = 54), only 49 significant DEGs were identified when comparing the 2-year follow up to baseline (adjusted *P* < .05 and |log_2_FoldChange|> 1) with 34 upregulated and 15 downregulated genes (Figure S4a). Deconvolution analysis showed no significant differences in estimated cell-type fractions between baseline and 2-year follow-up (Figure S4c). Together, these findings indicate that rectal mucosal transcriptomes and cellular compositions remain highly stable over a 2-year period in patients in clinical remission on anti-TNF therapy.

### 3.7. The inflammatory reaction in both withdrawal and maintenance groups upregulate genes that are targets for biologics and small molecule therapies

Despite its clinical efficacy, primary and secondary loss of response to anti‑TNF therapy occur in patients with UC. Several alternative treatments are available, including biologics targeting the IL-12/23 axis or integrin α4β7, and small molecule therapies such as JAK inhibitors.^5^^,^^38^ We therefore examined expression of genes and pathways corresponding to these therapeutic targets in patients who relapsed in the withdrawal group and in those with flare in the maintenance group.

Genes encoding p40 (subunit of IL-12 and IL-23) and p19 (subunit of IL-23), α4, and β7 were significantly upregulated in both relapse and flare patients. In addition, JAK-STAT signaling pathways were enriched in both groups (Figures 2D and 4D). A phase 2 study targeting anti-TNF-like cytokine 1A (TL1A) (encoded by *TNFSF15*) has reported clinical benefit in patients with moderate-to-severe UC,^39^ and consistent with this *TNFSF15* was increased in both groups. In contrast, the death receptor (DR)3 (encoded by *TNFRSF25*), shown to be highly upregulated in inflamed intestinal tissues, was downregulated in both groups (Figure 5C). Finally, given evidence for dysregulated mucosal B-cell responses in UC^40^^,^^41^ and a report of successful CD19 CAR T-cell therapy in a patient with multidrug-resistant UC,^42^ we examined B cell-related targets and found increased expression of *CD19* and *TNFSF13B* (encoding B-cell activating factor [BAFF]), a key regulator of B cell development, survival, and function, in both groups (Figure 5C).

Together, these findings suggest that multiple therapeutic target pathways are enriched both in relapse after anti-TNF withdrawal and flare during continued anti-TNF therapy, with potential implications for therapy class selection.

## 4. Discussion

In this study, we analyzed bulk RNA-seq data from longitudinal rectal biopsies of UC patients undergoing anti-TNF withdrawal and patients continuing anti-TNF therapy with a follow-up period of 2 years. We did not observe significant transcriptional differences at baseline between patients who relapsed and those who remained in remission after anti-TNF withdrawal. Patients who relapsed after treatment withdrawal and those who experienced flare on continued therapy displayed histologic and transcriptomic features characteristic of active UC inflammation. Notably, similar inflammatory gene expression profiles have been reported in previous studies to associate with non-response to anti-TNF therapy (eg, increased numbers of *THY1^+^ FAP^+^ PDPN^+^* activated fibroblasts). Therefore, these transcriptional profiles may not only reflect disease activity but also underlying differences in treatment responsiveness.^31^^,^^43^

The BIOSTOP study is the largest randomized clinical trial to date evaluating outcomes following anti-TNF withdrawal in UC patients in clinical and endoscopic remission. Consistent with previous randomized IBD anti-TNF de-escalation studies, approximately half of the patients who discontinued anti-TNF therapy relapsed during the 2-year follow-up, while the remaining patients maintained clinical remission.^13–17^ Importantly, most patients who relapsed regained clinical and endoscopic remission after reintroduction of anti-TNF therapy. Together, these findings support the notion that anti-TNF de-escalation may be a viable strategy for selected UC patients in endoscopically confirmed remission, with close disease monitoring.

A major aim of this study was to identify transcriptional features in mucosal biopsies that could distinguish patients who relapse from those who maintain long-term remission following anti-TNF withdrawal. However, neither differential gene expression analysis nor cell-type deconvolution revealed differences between these groups at baseline. The absence of detectable baseline differences suggests that relapse after anti-TNF withdrawal may not be driven by preexisting transcriptional or cellular states in the rectal mucosa prior to withdrawal. These findings suggest that mechanisms underlying relapse may involve dynamic changes that occur after treatment withdrawal.

Both relapsing patients in the withdrawal group and patients with flare on continued therapy showed histologic and transcriptomic hallmarks of active UC. We also observed enrichment of T cell-associated transcriptional signatures in both patient groups. Several DEGs identified in both comparisons were associated with CD8 T cell responses, together with enrichment of IL-17-related pathways. Although IL-17 is classically associated with Th17 cells, previous single cell RNA-seq studies on UC have shown expansion of IL-17-producing CD8 T cells, as well as inflammatory monocytes and fibroblasts, during active disease.^43^ These observations are consistent with our findings, and dysregulated T cell activation may be an important driver of inflammation in patients who lose response to anti-TNF therapy and those who relapse after anti-TNF withdrawal. While clinical trials of IL‑17 in IBD have failed to demonstrate efficacy and may exacerbate disease, our data indicate that a broader modulation of pathogenic T‑cell responses, such as targeting upstream cytokines (eg, IL‑23) or multiple Th17‑associated pathways, may provide a more effective therapeutic strategy for patient subgroups.^44^^,^^45^

The therapeutic landscape for UC has expanded substantially, with multiple biologics and small molecule drugs targeting distinct inflammatory pathways.^5^ In this study, we observed upregulation of several genes and pathways corresponding to approved and emerging therapeutic targets both in patients who relapsed after anti-TNF withdrawal and in those with flare on continued therapy. These findings support the clinical observation that patients who relapse on anti-TNF therapy may benefit from alternative treatments, such as IL-12/23 inhibitors, α4β7 integrin blockers, JAK inhibitors, or emerging approaches including B-cell-directed therapies.

Histologic assessment remains a cornerstone for evaluating disease activity in UC. We used RHI, which is heavily influenced by neutrophil infiltration in the tissue. Interestingly RHI correlated not only with neutrophil-associated transcriptional markers but also with estimated abundance of other inflammatory cell types, particularly monocytes/macrophages and activated fibroblasts. This suggests that RHI gives a good assessment of disease activity, beyond infiltration of neutrophils, and reflects the complex multicellular inflammatory environment in active UC.

A limitation of bulk mRNA-seq is the inability to fully disentangle cell-intrinsic transcriptional changes from those driven by shifts in cellular composition. Although we applied cell-type deconvolution to estimate relative cell-type proportions, this approach is constrained by quality and completeness of available reference datasets. To mitigate this, we used a single-cell reference closely matched to our cohort. At the same time, bulk RNA-seq offers advantages in robustness and sensitivity, particularly for detecting neutrophil-associated signals that are often underrepresented in single-cell RNA-seq due to the low mRNA content and technical fragility of neutrophils. In addition, our analysis was restricted to the transcriptomic layer, and molecular differences relevant to relapse prior to anti-TNF withdrawal may reside in other regulatory levels, such as epigenetic, proteomic, or metabolomic alterations. Furthermore, anti-drug antibody measurements were not available for all patients in the cohort and could therefore not be incorporated into the differential expression analyses. The sample size of this study may be another limitation, as a larger cohort could enable identification of predictive biomarker signatures.

In conclusion, this study provides a large, well-characterized longitudinal transcriptomic dataset of UC patients undergoing anti-TNF withdrawal or continued therapy. Although we did not identify predictive biomarkers for relapse after anti-TNF withdrawal, our findings offer important insights into the inflammatory mechanisms underlying relapse and flare. This dataset represents a valuable resource for future studies aimed at improving personalized treatment strategies for UC patients.

## Supplementary Material

jjag121_Supplementary_Data

## Data Availability

The sequencing data underlying the results in this article will be shared upon reasonable request to the corresponding author. All source code will be available on GitHub: https://github.com/JahnsenLab/UC-longitudinal-bulkRNA.
